# Apps for Covid-19 in Germany: assessment using the German Mobile App Rating Scale

**DOI:** 10.1093/jamiaopen/ooac082

**Published:** 2022-09-26

**Authors:** Felix Holl, Fabian Flemisch, Walter Swoboda, Johannes Schobel

**Affiliations:** DigiHealth Institute, Neu-Ulm University of Applied Sciences, Neu-Ulm, Germany; Institute for Medical Information Processing, Biometry, and Epidemiology, Ludwig Maximilian University of Munich, Munich, Germany; DigiHealth Institute, Neu-Ulm University of Applied Sciences, Neu-Ulm, Germany; DigiHealth Institute, Neu-Ulm University of Applied Sciences, Neu-Ulm, Germany; DigiHealth Institute, Neu-Ulm University of Applied Sciences, Neu-Ulm, Germany

**Keywords:** mHealth, COVID-19, evaluation study

## Abstract

**Objective:**

The purpose of this study is to provide an overview of apps to help control the spread of Covid-19 in Germany and rate them according to standardized instruments.

**Materials and methods:**

The Apple App Store and Google Play Store were systematically searched to identify apps dealing with Covid-19 in Germany. The German Mobile App Rating Scale (MARS-G) was used to independently assess app quality by 2 trained reviewers.

**Results:**

Overall, the quality of the 6 rated apps was good with a mean score of 4.15 (3.88–4.34). The best-rated apps were NINA app (4.34) and Corona Health App (4.29). The best-rated sections were functionality (4.40), aesthetic (4.25), and information (4.25). In contrast, the worst-rated section was engagement (3.63). Even though some of the apps were used by more people than others, there was no correlation between the MARS-G rating and app store rating. In addition, the MARS-G proved to be effective even with rating apps, which have different goals and methods to achieve them.

**Conclusions:**

To our knowledge, this is the first study that identified and evaluated German Covid-19 mobile health apps available in the German app stores. The review shows that despite the excellent quality in aspects like information and functionality, there is still a gap in the engagement section. To motivate more people to use the Covid-19 apps, new ideas are needed, besides more information and education about the functionality of the apps, to gain trust in app developers and raise the number of downloads.

## INTRODUCTION

Fueled by the wide availability of cellular networks and mobile phones, mobile health (mHealth) applications have become widely used to expand access to care and are being used to monitor communicable diseases.^1^^,^^2^ mHealth applications are already successfully used in low- and middle-income countries (LMICs) to prevent and control endemic infectious diseases like malaria and tuberculosis.^2^ Studies have also shown the potential for mHealth in contact tracing.^3^ In the 2014–2015 Ebola epidemic, mHealth apps also played an important role, for example, contact tracing and surveillance management, and many apps were developed and used.^4^

The Coronavirus disease 2019 (Covid-19) pandemic, which started in 2019, has put healthcare and public health systems around the globe under immense pressure. Measures to control the spread of the virus, such as social distancing, contact tracing, and information dissemination, have been put in place globally and have often been supported with mHealth apps.^5–7^ The main categories of apps for Covid-19 are: (1) tracking of the Covid-19 infection status, (2) population awareness, (3) prevention information, (4) remote assistance, and (5) information about treatment and services, and others. mHealth applications have been identified as a critical mechanism to control the spread of Covid-19, and a multitude of apps have been developed quickly.^8^^,^^9^ Apps for contact tracing of Covid-19 cases have been developed in several countries including Germany. A review has identified 17 different contact tracing apps from 15 countries.^10^ Yet many of them have not been fully evaluated to date.^11^ Apps have been used to monitor and study side effect tracking of the Covid-19 vaccines.^12^

Scholars have argued the importance of adhering to relevant regulatory guidelines and privacy compliance of such apps, especially given the short development cycles.^13^ A review and analysis of apps for Covid-19 in English using the Mobile Application Rating Scale (MARS) showed that all included mHealth apps scored acceptable (values > 3.0).^14^ While evidence about some of the Covid-19 apps in Germany have been published,^13^^,^^15^^,^^16^ no evidence evaluating all apps with the same tools exists yet.

This study aims to give an overview of apps for Covid-19 in Germany that are used country-wide and assess these apps using the German Mobile Application Rating Scale (MARS-G).^17^ The overview includes all nationally used apps for Covid-19 in Germany ranging from contact tracing, over information dissemination to vaccine side effect tracking. We excluded apps that were specifically developed for Covid-19 but only addressed common issues in healthcare delivery such as appointment scheduling.

## OBJECTIVE

The purpose of this study was to provide an overview of apps for Covid-19 in Germany and rate them according to standardized instruments.

## MATERIALS AND METHODS

### Search strategy

Both the Apple App Store and the Google Play Store were systematically searched across all categories to identify apps dealing with Covid-19 using the regional setting “Germany” and the following search terms: “Covid-19” OR “Corona” OR “Corona Warn App” OR “Corona App” OR “Robert-Koch-Institut” OR “Kontaktdaten” OR “Kontaktdaten verfolgen” OR “Covid-19 App” OR “Covid-19 App”. The last app store searches were performed on June 6, 2021.

The Robert Koch Institute is the German federal public health authority and was included in the search strategy. They had the governmental mandate and leadership in fighting the Covid-19 pandemic in Germany.

### Eligibility assessment and selection of apps

Only apps available for both the Android and iOS mobile operating systems were included in the analysis to reduce potential bias, including the potential digital divide.^18^ Smartphones running Android and iOS had a combined global market share of more than 99% in June 2021.^19^ Therefore, other operating systems were neglected in this study.

Apps meeting the following criteria were included in the analysis:

1. Standalone apps that do not require any additional devices, such as wearables, to be used.
2. Apps available in Germany (app store regional setting “German”) and the German language.

In addition, apps meeting the following criteria were excluded:

1. City or county-specific apps (eg, local or regional Covid-19 apps from cities and municipalities).
2. Apps in the pilot stage.
3. Scheduling apps for vaccinations and Covid-19 testing.

### Assessment

We applied the MARS-G to assess the applications included in the analysis. MARS-G is a translated and validated version of the Mobile App Rating Scale (MARS).^17^^,^^20^ The MARS has been increasingly adopted in recent years for evaluating mHealth apps as a standardized expert rating scale across different domains.^21–25^ MARS consists of 23 questions divided into the following 5 areas: engagement, functionality, aesthetics, information, and subjective Quality. Finally, 6 app-specific items provide additional information about the app in question. Each item is scored from 1 (inadequate) to 5 (excellent), and then a mean score is calculated for each section. The overall app quality score is derived from the mean values of the first 4 sections (engagement, functionality, aesthetics, and information). The subjective quality score is not included in the MARS-G score.

Two trained reviewers independently evaluated the quality of the included apps. Training was done as suggested by Messner et al.^17^ Both reviewers were in the final semester of an undergraduate program in a health sciences discipline. Thereby, each reviewer evaluated the app both on Android and iOS. Disagreements of greater than 2 points would have been discussed and resolved in a call, but did not occur.

Finally, the MARS-G scores were compared with the app store ratings to investigate similarities and differences between the results from a standardized expert tool with subjective end-user ratings.

## RESULTS

### App selection

**Figure 1.**
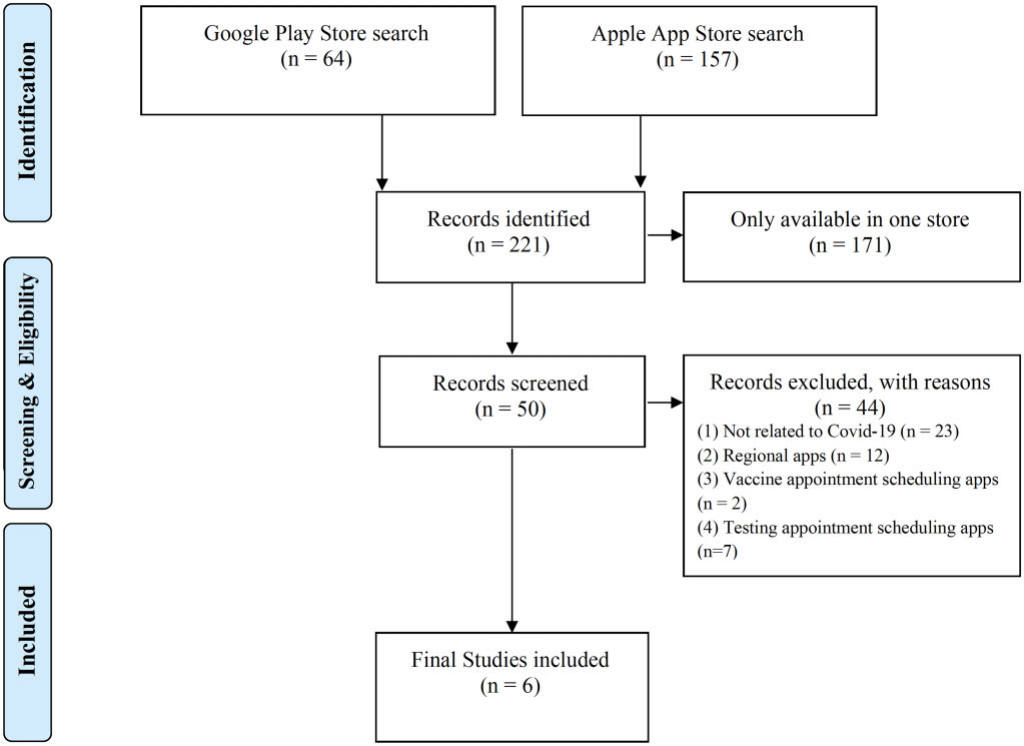
App identification and selection flow.

The last app store searches yielded 64 (Google Play Store) and 157 (Apple App Store) results. Fifty apps were available in both app stores. Forty-four apps were excluded because they did not meet the inclusion criteria. The excluded apps were local apps at the city or county level or testing or vaccine appointment scheduling. Apps from other German-speaking countries such as Austria and Switzerland were also excluded, as well as vaccine passport validation apps or apps unrelated to Covid-19. Six apps were extracted for analysis. All 6 apps are free of charge and do not offer in-app purchases. Figure 1 summarizes the identification, selection, and inclusion process.

### Characteristics and description of included apps

Table 1 gives an overview of the apps included in the study presented in this manuscript. It consists of the categories under which they are listed in the respective app stores, their target audience, and developer. In addition, the components of each app are listed.

**Table 1.** Apps included in the analysis with characteristics

App name	Apple App Store category	Google Play Store category	Target audience	Developer (as listed in app store)	Components^a^
Corona-Warn-App	Health & Fitness	Health & Fitness	Citizens	Robert Koch Institute	Mo, T, E
Corona Health App	Health & Fitness	Health & Fitness	Citizens	University Hospital Würzburg	D, Me, Mo, T
luca app	Utilities	Tools	Citizens	culture4life GmbH	D, Me, Mo, T
NINA app	Utilities	News & Magazines	Citizens	Federal Office for Civil Protection	I, E
SafeVac app	Medical	Medical	Citizens	Paul Ehrlich Institute	D, Me
STIKO app	Medical	Medical	Healthcare providers	Robert Koch Institute	I, E

a I: information; Mo: monitoring; Me: measurement; T: tracking information; E: education; D: data collection.

A detailed description of the apps included in the study can be found in Supplementary Appendix A.

Two apps, the SafeVac app and STIKO app, are vaccination apps. They were included because they are not vaccination scheduling apps, which were excluded. Four apps are listed in the App Store under “Medicine” or “Health & Fitness”. The luca app and NINA app are listed as “Utilities” in the App Store (iOS), but under “Tools” and “News & Magazines”, respectively, in the Google Play Store. The categorization is also the only difference between the iOS and Android apps stores. All apps were text- and graphics-based. In addition to texts and graphics, it is possible to play video clips in the STIKO app. The NINA app allowed the optional download of a notification sound that mimics a siren. Two of the apps (Corona Health and SafeVac) use questionnaires as the primary tool. However, only the Corona Health App includes a progress bar indicating the current processing state. The Corona-Warn-App is the only app that provides a diary function to keep track of encounters with others.

The target audience for all but one app is private individuals. Five of the 6 apps are designed to be used by people who have no prior medical knowledge. Only the STIKO app focuses on physicians or medical professionals as their main users.

The culture4life GmbH private company developed the luca app, public entities developed the remainder. One was executed by a university (Corona Health) and 4 by governmental organizations (Corona-Warn-App, NINA app, SafeVac, and STIKO app). The Robert Koch Institute is the developer of 2 apps (Corona-Warn-App and STIKO app).

The Corona-Warn-App, the Corona Health App, and the luca app are designed to facilitate monitoring and tracking. The Corona-Warn-App also focuses on the information and education aspect. The other 2 apps collect data to perform their main objectives. The Corona Health App is the only one of the apps that provides feedback. The NINA app and the STIKO app aim to provide information and education. The NINA app also provides tips and advice for extraordinary crises.

All the apps provide a privacy policy, an imprint, or a direct contact person. Notably, the Corona Health App is the only one that meets the standards of the Medical Device Regulation.^26^

Studies concerning the acceptance or user satisfaction have already been published on the NINA app, the Corona-Warn-App, and the luca app. The NINA app was published before the onset of Covid-19 because it serves as a long-term civil protection notification app in Germany.^27^ Results for the Corona-Warn-App and the luca app are only partially positive. The aspects include privacy and data security concerns, fueled by the general concerns of the public caused by the pandemic and restrictions imposed to control the pandemic.^16^^,^^28^

### MARS-G results

The overall scores of the 6 rated apps were good with a mean score of 4.15 (3.88–4.34). The best-rated apps were the NINA app (4.34) and Corona Health App (4.29). The lowest-rated apps were the STIKO app (3.88) and the SafeVac app (3.93).

The highest-rated sections were functionality (4.40), aesthetics (4.25), and information (4.25). The lowest-rated area was engagement (3.63). Detailed scores are shown in Table 2.

**Table 2.** MARS-G scores of the assessed applications

App name	Engagement	Functionality	Aesthetics	Information quality	Subjective quality	MARS-G score
Corona-Warn-App	**3.70** (3.60–3.80)	**4.50** (4.50–4.50)	**4.67** (4.67–4.67)	**3.93** (3.86–4.00)	**4.15** (4.0–4.3)	**4.20** (4.19–4.21)
Corona Health App	**3.60** (3.40–3.8)	**4.63** (4.50–4.75)	**4.33** (4.33–4.33)	**3.90** (3.80–4.00)	**3.63** (3.5–3.75)	**4.29** (4.21–4.37)
luca app	**3.80** (3.80–3.80)	**4.88** (4.75–5.00)	**4.33** (4.33–4.33)	**4.38** (4.25–4.50)	**3.63** (3.25–4.00)	**4.23** (4.17–4.28)
NINA app	**3.90** (3.80–4.00)	**4.50** (4.50–4.50)	**4.67** (4.67–4.67)	**4.25** (4.17–4.33)	**4.36** (4.25–4.50)	**4.34** (4.29–4.38)
SafeVac app	**3.20** (3.20–3.20)	**4.25** (4.25–4.25)	**3.84** (3.67–4.00)	**4.42** (4.33–4.50)	**3.75** (3.5–4.00)	**3.93** (3.91–3.95)
STIKO app	**3.60** (3.40–3.80)	**3.63** (3.50–3.75)	**3.67** (3.67–3.67)	**4.60** (4.60–4.60)	**4.25** (4.20–4.30)	**3.88** (3.79–3.96)

*Note*: Mean in bold and minimum and maximum in brackets.

The SafeVac app received the lowest rating from both reviewers in the engagement section. While the STIKO app and the SafeVac app are both apps that relate primarily to the area of vaccinations and received poor ratings in the subscales of engagement and aesthetics, the NINA app achieved the second-best score ever in a category with a 4.67 rating in aesthetics. Android apps were always given the same score as iOS apps by the respective reviewers, as there were no qualitative differences between the apps of the 2 platforms (Android and iOS) in the rating. However, there were variations in the rating among the reviewers. The only exception was the Corona Health App. Both reviewers experienced malfunctions of the app but on different operating systems.

**Figure 2.**
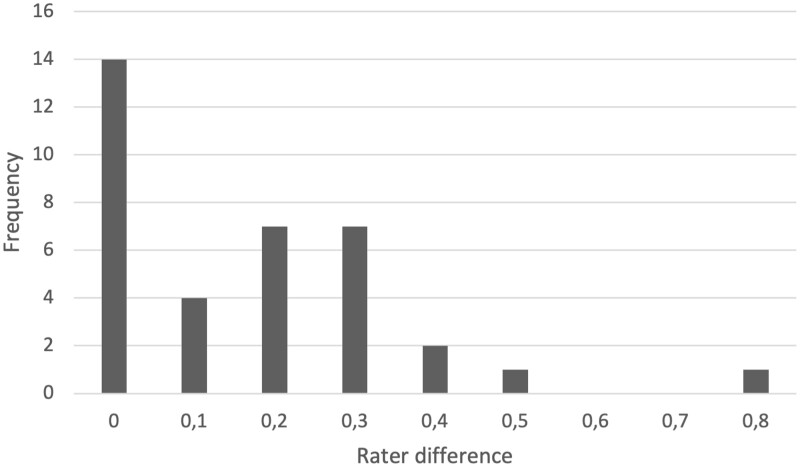
Frequency of rater differences.

The agreement between the 2 raters was high. The maximum difference in the total MARS-G score was –0.17 and –0.75 in individual scores, respectively. There was no difference in 14 out of 36 scores, and 25 were within a 0.2 difference. The rater differences are displayed in Figure 2.

## COMPARISON OF MARS-G SCORES AND APP STORE RATINGS

All apps in the Google Play Store were rated more frequently than in the Apple App Store. The STIKO app is the exception because it has nearly 10 times the reviews in the Apple App Store it has in the Google Play Store. Table 3 shows a clear difference between the number of reviews. The Corona-Warn-App has received the most reviews in the Google Play Store to date, with over 138 000 reviews by December 2021. This compares to 29 reviews for the Corona Health app in the Apple App Store. Since the download figures are not representative (Google Play Store) or are not available at all (Apple App Store), the number of reviews can be used to indicate how many people have downloaded the app. Due to the high level of public awareness of the Corona-Warn-App, it is not surprising that it was rated the most frequently by a clear margin. Overall, the app was rated twice as often by Android users as iOS users. In June 2021, the NINA app was the app with the most reviews (>20 000) in the Google Play Store, which can be attributed to the fact that this app existed before the pandemic and was rated over a more extended time. But by December 2021, the luca app had over 112 000 reviews in the Google Play Store, compared to just under 22 000 of the NINA app. Although the NINA app has the second most reviews in the Google Play Store of the 6 apps studied in June, 3 apps on the App Store (iOS) side have already received more reviews than the NINA app. This shows the varying popularity of the app by the operating system. Among Apple users, the luca app received the second most ratings by June 2021 and the most ratings by December 2021, and the rating increased from 2.9 to 4.4. Apart from the substantial increase of the luca app rating (by 1.5), the other rating was reasonably consistent between June and December 2021 with a maximum change of 0.3. While some of the store ratings showed high agreement with the MARS-G rating (Corona-Warn-App and app store rating both 4.2), there were also substantial discrepancies, both for apps with a large number and apps with a low number of reviews. No relevant correlation could be found between MARS-G scores and App Store ratings.

**Table 3.** MARS-G scores in comparison with app store ratings

App name	Mean MARS-G score	Google Play Store		Apple App Store	
		Rating (number of reviews)		Rating (number of reviews)	
		June 9, 2021	December 9, 2021	June 9, 2021	December 9, 2021
Corona-Warn-App	4.2	3.5 (127 035)	3.4 (138 913)	4.2 (61 009)	4.1 (64 266)
Corona Health App	4.29	4.1 (37)	4.0 (48)	3.8 (28)	3.8 (29)
luca app	4.23	4.3 (12 267)	4.2 (112 087)	2.9 (5035)	4.4 (388 505)
NINA app	4.34	3.4 (20 138)	3.2 (21 966)	2.7 (2586)	2.6 (3733)
SafeVac app	3.93	3.3 (1853)	2.9 (2875)	3.2 (825)	2.9 (1516)
STIKO app	3.88	4.1 (568)	4.4 (969)	4.6 (4507)	4.6 (8566)

## DISCUSSION

### Principal results

mHealth apps have been used in LMICs for several years to help track infectious diseases and control the spread of diseases.^2^ With the Covid-19 pandemic, mHealth apps have also become a widely used tool in high-income countries to help contain the global pandemic. While mHealth apps before the Covid-19 pandemic were developed only for specific diseases with small numbers of people affected, 2020 is the first time that health apps developed for the entire population have emerged. Some apps were introduced to citizens through large advertising campaigns, with only moderate success.

Several apps for Covid-19 have been developed and released in Germany. The high ratings of all apps across most categories show that all apps are logical, structured, and state of the art. From a technical point of view, each evaluated app can execute the functions for which the app was initially developed. The high quality is visible in the good average score of 4.15, evident in all apps. Nevertheless, the STIKO app shows significantly longer loading times compared to all other apps, which is why it was rated the worst here in the functionality aspect. It is also noticeable that all apps require Internet access to be fully operational. However, there are differences in whether the Internet connection must be maintained or only present for updates in case of news. A particular focus in the apps evaluated was on information, education, and prevention of the disease. Behavior modification or memory aids were not the focus of the apps. The luca app achieved the best average result of all subscales in functionality with a rating of 4.8 out of 5.0. Only the NINA app and the STIKO app received higher ratings in subjective quality than in the MARS-G score. The remaining apps were rated better based on the rating sheet than they appeared to be based on subjective aspects. The reviewers’ agreement was high (maximum deviation of 0.4) for the overall MARS-G score and (maximum deviation of 0.17) for the individual scales. Accordingly, high interrater reliability can be assumed here; as seen from the Supplementary Appendix, the assessments in the 4 main categories deviated from each other only 6 times by a factor of 2 or more. SafeVac scored low in the engagement category because of the app’s lack of customizability.

The overall quality of the apps is rated high, despite the short development time of the apps. Federal institutions or public academic institutions developed all but one application. This fact could explain the high information quality rating. The low scores in the engagement section show that there is still room for improvement to increase the uptake of Covid-19 apps in Germany.

Privacy issues are an important factor for Covid-19 apps. The Corona-Warn-App applies the most privacy sensitive approach by only storing the contact information locally in anonymous form. This approach is only applied by few other countries.^10^ However, the usage of the CWA is low, and the effectiveness is therefore limited. On the other hand, the luca is widely used despite its privacy concern.

No correlations between MARS-G rating and app store ratings were demonstrated. These results show an objective view of Covid-19 apps in Germany. No previous studies evaluating Covid-19 apps in Germany have been published yet. However, the robustness of these findings is limited as the MARS-G rating is an evaluation at one point in time, while the app store ratings are accumulated over time. While the rating of all but one app was relatively consistent over time with a maximum change in the ratings of 0.3 between June and December 2021, the limitation is highlighted by the fact that the luca app had a rating of 2.9 in June 2021 in the Apple App Store and the rating increased to 4.4 by December 2021.

### Limitations

This study is limited because apps were searched and downloaded on June 6, 2021. Given the dynamic of the Covid-19 situation, apps get updated often. In addition, the luca app received a high rating but has been heavily criticized for its security vulnerability, which is not reflected in the rating.^29^ Data on the number of downloads were only available for the Corona-Warn-App and the luca app but not the other 4 apps.

### Comparison with prior work

To our knowledge, this is the first study that identified and assessed Covid-19 apps available in app stores in Germany. Our findings are comparable with ratings of apps for Covid-19 using MARS in other settings and languages.^14^^,^^30^ Our findings about low scores in the engagement section are similar to prior work.^14^

## CONCLUSION

Despite their short development times, the apps used to help control the spread of Covid-19 in Germany have an overall high quality. The study has shown that there is still room for improvement in the engagement section despite the good quality in aspects such as information and functionality. To improve the apps in the engagement section, it may be useful to engage end-users and user-experience experts in future development. In this way, both appealing and high-quality apps could be developed. It is also important to sensitize users about the privacy of the Covid-19 apps to improve usage of those apps with good privacy compliance such as the CWA.

The findings from the study highlight the feasibility of quickly developing mHealth apps as a tool when responding to rapidly emerging health threads like a pandemic. It is important to pay attention to an app design that engages the user to use the app to ensure high utilization. Especially for communicable health treads, where contact tracing is important, high utilization is key.

## FUNDING

This research received no specific grant from any funding agency in the public, commercial, or not-for-profit sectors.

## AUTHOR CONTRIBUTIONS

Conceptualization, FH; methodology, FF and FH; software, FF; validation, FH; formal analysis, FF; investigation FF; resources, FF and FH; data curation, FF; writing—original draft preparation, FH; writing—review and editing, WS and JS; visualization, FF and FH; supervision, JS. All authors have read and agreed to the published version of the manuscript.

## SUPPLEMENTARY MATERIAL

Supplementary material is available at *JAMIA Open* online.

## CONFLICT OF INTEREST STATEMENT

JS was involved in the conceptualization and development of the Corona Health app. He supervised the presented work and was not involved in the data analysis.

## Supplementary Material

ooac082_Supplementary_Data

## Data Availability

Data are available on request.
