# Supplementary material for: Apps for Covid-19 in Germany: assessment using the German Mobile App Rating Scale
Source: JAMIA Open. 2022 Sep 26;5(4):ooac082. doi: 10.1093/jamiaopen/ooac082 (PMC9543402; doi:10.1093/jamiaopen/ooac082)
Supplement: ooac082_Supplementary_Data [file ooac082_supplementary_data.docx]

#### **DESCRIPTION OF THE APPS INCLUDED IN THE STUDY**

#### **Corona-Warn-App**

The German government released the Corona-Warn-App (CWA) through the Robert-Koch Institute, the German federal public health authority, as the official Covid-19 contact tracing app in Germany on June 16, 2020. The CWA had already been downloaded over 34 million times by the end of September 2021 and was developed by Deutsche Telekom and SAP AG.[1] The app was promoted as an effective way to track contacts and thus help contain the Covid-19 pandemic in Germany.[2] It captures encounters relying on Bluetooth connections. An anonymous ID is stored on smartphones if two devices have been less than two meters from each other for more than 15 minutes. If a person tests positive for the virus and the test result is published in the app, all persons connected to the infected person's smartphone receive a warning ("increased risk"). If there was no contact with an infected person, the status "low risk" is displayed. Individuals who receive a warning message can move into isolation faster to prevent further spread and help break chains of infection. By providing warnings, the CWA can assist public health departments in following up on contacts. The decentralized data storage and tracking (tracing) show the high priority to data protection during development. The app has been steadily developed further. Since December 2020, the app works across country borders, and a contact diary function has been introduced.

Scholar have found the CWA to have limited effectiveness.[3] This limitation is not caused by the conception of the app, but the insufficient distribution among the population (e.g., hard to reach population as not using the app), despite high download numbers. The CWA is only effective with high usage rates and homogeneous distribution across the population.[2]

#### **luca App**

The luca app was developed by neXenio GmbH and is maintained by culture4life GmbH. neXenio GmbH is a spin-off from the Hasso Plattner Institute, a leading German academic information technology institute. The conceptualization of the luca app was supported by a group of creative artists, mainly the band Die Fantastischen Vier.[4] The luca app was released in April 2021. With the help of this app, the contact details of attendees at events, bars, and restaurants can quickly be recorded. The luca app also offers the function to collect contact details for private meetings. The focus here, however, is on active user participation. Users can register with their names and contact details in the smartphone app. When users enter a venue, where contact details of attendees need to be recorded, they scan the venue’s luca app QR code (usually posted at the entrance), and their visit details are recorded in the central luca database. Without the luca app, visitors must leave their contact details in paper-based record sheets. Some venues use proprietary web-based contact detail recoding systems as an alternative to the paper-based records and the luca app.[5] The app is designed to collect whereabouts and personal data, reducing paperwork for venue owners and event organizers (who must track contacts). If a person tests positive for Covid-19, they can submit the data to the health department. By connecting to the health departments, they can then contact anyone who was checked in at the same place at the same time. The app is already linked to health offices in 13 states and has been downloaded by 20 million users by July 2021.[6,7] Despite the great willingness to download the app, the central data storage has met with criticism from data protectionists.[8] Data protection gaps, security issues, and a "questionable allocation practice" are also cited as points of criticism. [9] In a study examining the acceptance of the luca app, 25% of respondents stated that they refrained from using the app due to concerns about data protection.[10]

#### **NINA App**

The Emergency Information and News App (NINA) of the Federal Office of Civil Protection and Disaster Assistance warns users in Germany in dangerous situations (i.e., due to natural events). A map provides users with an overview of the current dangerous situation in Germany. In addition, warnings from the German Weather Service (severe weather), the Flood Control Center (floods), and the Disaster Control Center (significant fires, hazardous material spread) are displayed. By voluntarily activating location tracking, the user receives a push message when a warning is issued for an area that affects their location.[11] In addition to the current situation, users receive useful information on how to act in the event of an emergency or disaster.[12] The app also provides information on current and locally applicable regulations on the Covid-19 situation and includes general information on the risk posed by the pandemic. The NINA app was released in 2015 and was not developed due to the Covid-19 pandemic.[13]

#### **Corona Health App**

Users of the Corona Health app can participate in five different scientific surveys. With these surveys, scientists from the universities of Würzburg, Regensburg, Ulm, and the Robert Koch Institute want to determine the effects of the pandemic on the physical and mental health of adults and children.[14] Users can choose which study they would like to participate in. Currently, the mHealth app offers studies dealing with "mental health for adults (18 years and older)," "physical health for adults (18 years and older)," "mental health for adolescents (12 to 17 years and older)," "recognizing stress for adults (18 years and older)," and a study of the "Network University Medicine: Compass Project on the Acceptance of Pandemic Apps (18 years and older)." Additionally, "News" about the Corona Pandemic is displayed. After a more extensive initial survey, study participants are asked at regular intervals (weekly or biweekly) to answer a short questionnaire describing the current situation and changes. By collecting passive communication data in the background, sending the location, and answering the questionnaire, the app aims to collect the user's activities and communication behavior, thus enabling a better understanding for researchers.[15] The app differs from tracking apps, as the location is only determined to be within 11 km and is therefore not suitable for location tracking. A study by the developers confirms the usefulness of this app.[14] The Corona Health app, developed in 2020, is the only app in this selection that complies with the requirements for a medical device law as it is certified according to the IEC 62304, IEC 82304, and GAMP 5 standards. However, the app does not have CE certification and is not officially classified as a medical device.[14] The Corona Health App was developed by multiple universities with two private companies and the RKI.[16]

#### **SafeVac App**

The SafeVac app aims to record the tolerability and safety of Covid-19 vaccinations. It was developed by the Paul Ehrlich Institute and funded by the German Federal Ministry of Health. The technical implementation was carried out by Materna Information & Communications S.E. The aim of the cohort study using an app is the intensive monitoring and recording of adverse vaccination reactions. The app is intended to serve as an immunization diary in which the user documents the occurrence and non-occurrence of vaccination reactions.[17] To achieve this, users are asked about their health status at set points in time. The user must know the vaccine name and batch number to be able to use the app. In addition, no more than 48 hours may have passed since the time of vaccination. A further prerequisite for use is a minimum age of 18 years. Participation is voluntary and can be terminated at any time.[17] The SafeVac App was originally developed as the SafeVac App 1.0. This version is no longer available, and the app is currently offered as SafeVac App 2.0 in the App Store. The SafeVac App 1.0 focused on annual influenza vaccinations and was developed in 2018 and discontinued in 2019.[18] In the new version, the focus is on Corona vaccines. A study of the app's first version confirmed its suitability for a supplemental recording of adverse vaccination events.[19] SafeVac 2.0 was developed in 2020.[20] However, no evaluations are available for the new app version (SafeVac 2.0).

#### **STIKO App**

The STIKO app was developed by the Robert Koch Institute for physicians and healthcare professionals in collaboration with Börm Bruckmeier Verlag. The aim is to support healthcare professionals with questions about vaccinations in practice. For this purpose, "specialist information on all vaccines, answers to frequently asked questions about vaccinations, and the RKI guides on vaccine-preventable diseases are available".[21] The app is intended to help users (i.e., physicians) quickly obtain the relevant information on the corresponding vaccination. The recommendations of the Standing Commission on Vaccination (STIKO) provide the basis of the information here. The STIKO app was initially developed in 2016.[22] The STIKO app was updated accordingly to fit the new requirements and provide information in the context of the Covid-19 pandemic. In addition to news on the Corona pandemic, the app also offers information material, explanatory films, specialist information on vaccines and pathogens, a vaccination calendar, and an individual vaccination check for standard and follow-up vaccinations.[21]

**References**

1 Statista. Corona-Warn-App: Downloads in Deutschland 2021. https://de.statista.com/statistik/daten/studie/1125951/umfrage/downloads-der-corona-warn-app/ (accessed 22 Nov 2021).

2 Blom AG, Wenz A, Cornesse C, *et al.* Barriers to the Large-Scale Adoption of a COVID-19 Contact Tracing App in Germany: Survey Study. *J Med Internet Res* 2021;**23**. doi:10.2196/23362

3 Urban M. „Die Hoffnung, informiert zu sein“. Effekte der Corona-Warn-App. *Pra¨vention Und Gesundheitsfo¨rderung* 2021;:1. doi:10.1007/S11553-021-00854-9

4 Luca App. Über uns. https://www.luca-app.de/uber-uns/ (accessed 28 Jan 2022).

5 Köver C, Fanta A. Digitale Kontaktverfolgung: Mehr als 20 Millionen Euro für Luca. 2021.https://netzpolitik.org/2021/digitale-kontaktverfolgung-fast-20-millionen-euro-fuer-luca/ (accessed 7 Dec 2021).

6 luca app – Apps bei Google Play. https://play.google.com/store/apps/details?id=de.culture4life.luca&hl=de&gl=US (accessed 7 Dec 2021).

7 Ärzteblatt. Luca-App durchbricht Schwelle von 20 Millionen Nutzern. https://www.aerzteblatt.de/nachrichten/125196/Luca-App-durchbricht-Schwelle-von-20-Millionen-Nutzern (accessed 7 Dec 2021).

8 Gemeinsame Stellungname zur digitalen Kontaktnachverfolgung. https://digikoletter.github.io/ (accessed 7 Dec 2021).

9 Chaos Computer Club. Luca-App: CCC fordert Bundesnotbremse. https://www.ccc.de/de/updates/2021/luca-app-ccc-fordert-bundesnotbremse (accessed 7 Dec 2021).

10 Munzert S, Selb P, Gohdes A, *et al.* Tracking and promoting the usage of a COVID-19 contact tracing app. *Nature Human Behaviour* 2021;**5**:247–55. doi:10.1038/s41562-020-01044-x

11 Grinko M, Kaufhold M-A, Reuter C. Adoption, Use and Diffusion of Crisis Apps in Germany: A Representative Survey. *Proceedings of Mensch und Computer 2019* 2019;:12. doi:10.1145/3340764

12 Kotthaus C, Ludwig T, Pipek V. Persuasive System Design Analysis of Mobile Warning Apps for Citizens. In: *Adjunct Proceedings of the 11th International Conference on Persuasive Technology, (Persuasive ’16)*. 2016.

13 Bundesamt für Bevölkerungsschutz und Katastrophenhilfe. Warn-App NINA. https://www.bbk.bund.de/DE/Warnung-Vorsorge/Warn-App-NINA/warn-app-nina_node.html (accessed 28 Nov 2021).

14 Beierle F, Schobel J, Vogel C, *et al.* Corona Health—A Study- and Sensor-Based Mobile App Platform Exploring Aspects of the COVID-19 Pandemic. *International Journal of Environmental Research and Public Health* 2021;**18**:7395. doi:10.3390/IJERPH18147395

15 Beierle F, Dhakal U, Cohrdes C, *et al.* Public perception of the German COVID-19 contact-tracing app corona-warn-app. *Proceedings - IEEE Symposium on Computer-Based Medical Systems* 2021;**2021-June**:342–7. doi:10.1109/CBMS52027.2021.00031

16 Corona Health App. Team. https://www.corona-health.net/en/#team (accessed 28 Jan 2022).

17 Oberle D, Mentzer D, Weber G. Befragung zur Verträglichkeit der Impfstoffe gegen das neue Coronavirus (SARS-CoV-2) mittels Smartphone-App SafeVac 2.0. *Bulletin of Drug Safety* 2020;**4**.https://www.pei.de/SharedDocs/Downloads/EN/newsroom-en/pharmacovigilance-bulletin/single-articles/2020-safevac-app-en.pdf?__blob=publicationFile&v=3 (accessed 28 Nov 2021).

18 Mentzer D, Keller-Stanislawski B, Ott JJ, *et al.* Digitalisierung: Epidemiologische Studie mit App- basierter Erfassung von Symptomen nach betriebsärztlicher Influenzaimpfung. *Bulletin für Arzneimittelsicherheit* 2018;**3**:30–2.

19 Krause G, Ott JJ, Keller-Stanislawski B, *et al.* Erfassung unerwünschter Wirkungen der Influenzaimpfung mittels mobiler Technologien (Apps) im Rahmen einer epidemiologischen Pilotstudie unter Einbindung von Betriebsärzten (VigilVacMobile). https://www.bundesgesundheitsministerium.de/fileadmin/Dateien/5_Publikationen/Gesundheit/Berichte/Abschlussbericht_VigilVacMobile_HZI_bf.pdf (accessed 28 Nov 2021).

20 Oberle D, Mentzer D, Weber G. Befragung zur Verträglichkeit der Impfstoffe gegen das neue Coronavirus (SARS-CoV-2) mittels Smartphone-App SafeVac 2.0. *Bulletin für Arzneimittelsicherheit* 2020;**4**:27–31.

21 Robert-Koch-Institut. STIKO@rki - die neu gestaltete Impf-App für ÄrztInnen und Gesundheitsfachpersonal. https://www.rki.de/DE/Content/Kommissionen/STIKO/App/STIKO-App_node.html (accessed 28 Nov 2021).

22 Bödeker B. Ein Jahr STIKO@rki-App: Eine kleine Erfolgsgeschichte. *Epidemiologisches Bulletin* 2017;**40**:457–61. doi:10.17886/EpiBull-2017-052
